# Persistent B Cell Depletion After Rituximab for Autoimmune and Glomerular Diseases: A Case Series

**DOI:** 10.1016/j.ekir.2025.02.002

**Published:** 2025-02-07

**Authors:** Orhan Efe, Gabriel Sauvage, Anushya Jeyabalan, Ayman Al Jurdi, Harish S. Seethapathy, Katherine Cosgrove, Frank B. Cortazar, Karen A. Laliberte, Reza Zonozi, John L. Niles

**Affiliations:** 1 Vasculitis and Glomerulonephritis Center, Massachusetts General Hospital, Boston, Massachusetts, USA; 2 Division of Nephrology, Massachusetts General Hospital, Boston, Massachusetts, USA; 3 Harvard Medical School, Boston, Massachusetts, USA; 4 New York Nephrology Vasculitis and Glomerular Center, Saint Peter's Hospital-Albany, Albany, New York, USA; 5 Nephrology Associates of Northern Virginia, Fairfax, Virginia, USA; 6 Inova Fairfax Hospital, Falls Church, Virginia, USA

**Keywords:** complications of rituximab, persistent B cell depletion, prolonged B cell depletion, rituximab

## Abstract

**Introduction:**

Persistent B cell depletion is a rare complication of rituximab treatment, and its clinical implications are unknown.

**Methods:**

This retrospective case series included patients with glomerular and autoimmune diseases who developed persistent B cell depletion (< 5 CD19^+^CD20^+^ cells/μl persisting for > 2 years) after the last rituximab dose.

**Results:**

Among 1519 patients who received rituximab, 2% (*n* = 30) had persistent B cell depletion. The frequencies of persistent B cell depletion were 2.5% (22 of 878), 2.4% (2 of 82), and 0.8% (1 of 114) in antineutrophil cytoplasmic autoantibody (ANCA)-associated vasculitis (AAV), systemic lupus erythematosus (SLE) or lupus nephritis, and podocytopathies, respectively. The remaining patients had ANCA-negative vasculitis (*n* = 2), anti-glomerular basement membrane disease (*n* = 1), Behcet’s disease (*n* = 1), and polymyositis (*n* = 1). The median age was 64.5 (interquartile range [IQR]: 44–77) years. Before the last dose of rituximab, all patients except 2 used cytotoxic agents, often prolonged (> 1 year) or recycling courses, and 60% (18 of 30) received a period of long-term maintenance steroids. By 4 years after the last rituximab dose, only 30% had B cell repopulation. In those who experienced B cell repopulation, B cell counts remained very low, at a median of 7(6–15) cells/μl at the last follow-up. After the last rituximab dose, 83% (23 of 30) had sustained disease remission. Late-onset neutropenia, recurrent infections, and severe infections occurred in 23% (7 of 30), 47% (14 of 30), and 57% (17 of 57), respectively. Of the patients, 23% (7 of 30) required immunoglobulin replacement, and 30% (9 of 30) died, mostly from complications of chronic diseases.

**Conclusion:**

Persistent B cell depletion is a rare complication of rituximab treatment, mostly affecting patients with exposure(s) to cytotoxic therapies for recurrent diseases. It is characterized by prolonged disease remission and increased infection risk.

## Graphical abstract

**Figure:**
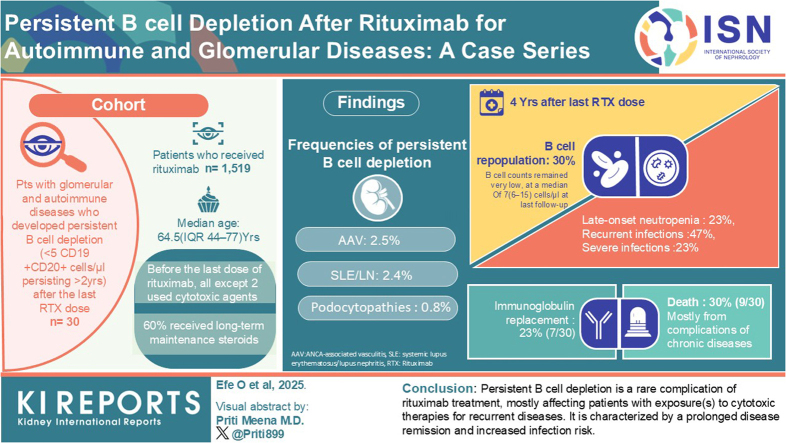

See Commentary on Page 1315

Historically, autoantibody-driven autoimmune diseases such as AAV^1^^,^^2^ and glomerular diseases such as membranous nephropathy^3^ and focal segmental glomerulosclerosis^4^, ^5^ have been associated with high rates of debilitating injury and death, both because of disease activity and treatment-related toxicities. Rituximab, a chimeric monoclonal anti-CD20 antibody that depletes a broad array of B cells from pre–B cells to memory B cells,^6^ has emerged as a mainstay treatment for many autoantibody-driven diseases because of its ability to induce and maintain remission with a favorable safety profile.^6^, ^7^, ^8^, ^9^, ^10^ Peripheral B cells usually remain depleted for 6 to 12 months after a single course of rituximab.^1^^,^^11^, ^12^, ^13^ Maintenance rituximab doses are typically administered on a fixed schedule (e.g., every 6 months) or at extended intervals based on B cell recovery, disease biomarkers, or disease activity.^13^, ^14^, ^15^

Nevertheless, rituximab treatment is not without its own risks, and the use of extended courses is revealing new risks. The most common adverse effects of rituximab include infusion reactions, hypogammaglobulinemia, infections, and late-onset neutropenia.^16^^,^^17^ A rare complication of rituximab use is abnormally prolonged B cell depletion lasting years after rituximab treatment, which we define as a new phenomenon, “persistent B cell depletion.” A few studies have shown prolonged B cell depletion in patients with AAV^18^, ^19^, ^20^ and few case reports exist in other diseases.^21^ However, the frequency, risk factors, and clinical consequences of this complication are relatively unknown.

In this retrospective study, we investigated the frequency of persistent B cell depletion following rituximab treatment among 1519 patients who received rituximab for various autoimmune and glomerular diseases. We present the patient characteristics, potential predisposing factors, and clinical implications of persistent B cell depletion and provide insight into managing its complications such as infections and late-onset neutropenia, with a long follow-up.

## Methods

### Study Design and Patients

This single-center retrospective cohort study included patients with autoimmune and glomerular diseases who had persistent B cell depletion after receiving rituximab at the Vasculitis and Glomerulonephritis Center at Massachusetts General Hospital in Boston, Massachusetts, between January 01, 2003 and December 30, 2021. Persistent B cell depletion was defined as a total CD19^+^CD20^+^ cell count of < 5 cells/μl lasting for more than 2 years from the last rituximab dose based on our clinical experience and the MAINTANCAVAS trial, which evaluated the rituximab maintenance strategy for B cell return versus ANCA titer increase, where the median time to B cell repopulation after the last rituximab dose was 9.44 months (IQR: 8.94–12.13), and all patients had B cell repopulation within the first 2 years. Patients were included if they were aged > 18 years, received at least 1 dose of rituximab for the treatment of autoimmune or glomerular diseases, and met the criteria for persistent B cell depletion. Patients were excluded if they had no peripheral flow cytometry performed or had < 2 years of B cell depletion from the last rituximab dose. B cell counts were measured using standard flow cytometry with fluorescent-labeled monoclonal antibodies against the following cell surface markers: CD3, CD4, CD5, CD8, CD19, and CD20 at Massachusetts General Hospital Laboratories. All data were collected from the electronic medical records. The study was approved by the Mass General Brigham Institutional Review Board (protocol number 2007P000933). The requirement for informed consent was waived by the ethics committee because of the retrospective nature of the study.

### Treatment Regimen

Patients included in this study may have been on maintenance immunosuppression and transitioned to rituximab or started on rituximab as part of an induction therapy for a new disease or clinical relapse. Our "standard” rituximab treatment has typically consisted of two 1000 mg i.v. doses separated by 2 to 4 weeks for induction, followed by a single 1000 mg i.v. dose every 4 to 6 months to start, as described previously.^10^^,^^22^ When rituximab treatment continued for > 2 years, dosing intervals were often extended to allow B cell repopulation. For membranous nephritis and minimal change disease, rituximab was usually discontinued after 2 years unless the patient relapsed. B cell (CD19^+^CD20^+^) counts were typically measured before each rituximab dose or at 3- to 6-month intervals after stopping rituximab.

### Patient Characteristics and Predisposing Factors

We analyzed patient characteristics, including age, biological gender, underlying disease, duration of disease before rituximab treatment, number of rituximab doses, cumulative rituximab dose, previous and concurrent exposure to other immunosuppressants, disease control, and kidney function.

### Outcomes

The primary outcome measure was the duration of B cell depletion, calculated for each patient from the date of the last rituximab dose through the last documented date of B cell depletion before B cell repopulation. If documented B cell repopulation was unavailable, we censored the period on the last date of documented B cell depletion. Secondary outcomes included disease relapse requiring additional immunosuppression, including recycling of steroids, serum ANCA levels in patients with AAV, patient survival, serum Ig levels, use of IgG replacement, infections, and other complications of rituximab treatment such as late-onset neutropenia and inflammatory vaginitis.

### Definitions

One dose of rituximab corresponded to 1 i.v. infusion. Long-term maintenance steroid treatment was defined as the use of prednisone ≥ 5 mg/d or equivalent for > 1 year. The Chronic Kidney Disease-Epidemiology Collaboration equation was used to calculate the estimated glomerular filtration rate at the time of the last rituximab dose. Hypogammaglobulinemia was defined as IgG < 614, IgA < 69, or IgM < 53 mg/dl as per our center’s cut-off values. Late-onset neutropenia was defined as an absolute neutrophil count < 1000 cells/μl during the period of B cell depletion without an alternative identifiable etiology. Recurrent infection was defined as ≥ 3 infections annually, except *≥* 5 courses for upper respiratory tract infections. A severe infection was defined as an infection requiring hospitalization or i.v. antibiotic treatment. Inflammatory vaginitis was diagnosed when vaginal cultures were negative, and vaginitis was resistant to antibacterial and antifungal treatments.

### Statistical Analysis

Values for continuous variables were presented as the median (IQR) or as the mean ± SD, as appropriate. Values for categorical variables were presented as frequencies and percentages. Differences between continuous variables were assessed using *t* tests, and categorical variables were assessed using chi-square or Fisher exact tests, as appropriate. All comparisons were 2-tailed, with *P* < 0.05 considered statistically significant. The analyses were performed using STATA version 15 (StataCorp), and graphs were created using GraphPad Prism (Boston, MA).

## Results

### Patient Demographics and Clinical Characteristics Until the Date of Last Rituximab Dose

During the study period, 1519 patients received rituximab at our center, most commonly for AAV (*n* = 878, 57.8%) (Supplementary Table S1), and 2.0% (*n* = 30) developed persistent B cell depletion. The frequencies of patients who developed persistent B cell depletion included 2.5% (22 of 878) in AAV, 2.4% (2 of 82) in SLE or lupus nephritis, 0.8% (1 of 114) in minimal change disease or focal segmental glomerulosclerosis, and 0.0% (0 of 133) in membranous nephropathy. The remainder of diagnoses included ANCA-negative vasculitis (2 of 37), anti–glomerular basement membrane disease (1 of 6), Behcet’s disease (1 of 1), and polymyositis (1 of 2); however, the total number of patients receiving B cell depletion therapy was too low to estimate its frequency in these diseases. Patient characteristics are summarized in Table 1. The median age was 64.5 (IQR 44–77) years and 73% (22 of 30) had AAV. The median estimated glomerular filtration rate was 65 (IQR 37–85) ml/min per 1.73 m^2^, including 3 patients who received kidney transplants. Two patients were on dialysis. Individual patient information is depicted in Supplementary Table S2.

**Table 1:** Demographics and clinical features before rituximab initiation (*n* = 30)

Characteristics	Median (IQR) or *n* (%)
Age at last rituximab dose	64.5 (44–77)
Female	17 (57)
Race	
White	27 (90)
Black	1 (3)
Hispanic	1 (3)
Asian	1 (3)
Primary disease	
ANCA-associated vasculitis (AAV)	22 (73)
MPO-AAV	18 (60)
PR3-AAV	4 (13)
ANCA-negative vasculitis	2 (7)
Lupus nephritis	2 (7)
FSGS	1 (3)
Anti-GBM disease	1 (3)
Bechet’s disease	1 (3)
Polymyositis	1 (3)
Presence of other autoimmune diseases or hematological malignancies (Supplementary Table S2)	9 (30)
Estimated glomerular filtration rate (ml/min per 1.73 m^2^)	65 (37–85)
Number of patients who experienced relapse before rituximab	17 (57)
1 – 2 relapses	5 (29)
3 – 4 relapses	2 (12)
5 or more relapses	10 (59)
Exposure to cytotoxic therapies preceding or concurrent with rituximab initiation	29 (97)
Cyclophosphamide	23 (77)
Azathioprine	13 (43)
Mycophenolate mofetil	9 (30)
Methotrexate	3 (10)
Others^a^	5 (16)
Long-term maintenance steroids (≥1 yr) preceding or concurrent with rituximab initiation	18 (60)
Clinical features during the period of rituximab doses	
Baseline B cell count (cells/μL, before first Rituximab)	120 (82–247)
Time from diagnosis to first rituximab (yrs)	1.1 (0.7–6.6)
Time from first rituximab to last rituximab (yrs)	3.5 (2.5–5.1)
Number of rituximab doses	11 (8–13)
Cumulative rituximab exposure (mg)	10,500 (8000–12,000)
Patients who experienced relapse requiring repeat rituximab courses	9 (30)
1 – 2 relapses	7 (78)
3 – 4 relapses	2 (18)
5 or more relapses	0 (0)

AAV, ANCA-associated vasculitis; ANCA, antineutrophil cytoplasmic autoantibody; FSGS, focal segmental glomerulosclerosis; IQR, interquartile range; MPO, myeloperoxidase; PR3, proteinase 3.

a Others include calcineurin inhibitors, anti-thymocyte globulin, tocilizumab, anti-TNF agents, avacopan, and plasma exchange.

Fifty-seven percent (17 of 30) of the patients failed to respond to cytotoxic immunosuppressive agents before rituximab, and 60% (10 of 17) of those had > 5 relapses (Table 1). All except 2 patients were exposed to immunosuppressive agents other than rituximab, mostly cyclophosphamide, azathioprine, and mycophenolate mofetil, often prolonged (> 1 year) or repeated courses (Supplementary Table S2), and 60% (18 of 30) had received long-term maintenance steroids preceding or concurrent with rituximab (Table 1).

Patient characteristics during the period of rituximab treatment are summarized in Table 1. The median time from diagnosis to rituximab initiation was 1.1 (0.7–6.6) years. The patients received a median cumulative rituximab dose of 10,500 (IQR: 8000–12,000) mg over a median of 3.5 (IQR: 2.5–5.1) years. The total duration of immunosuppression until the last dose of rituximab was 7.0 (IQR: 3.6–10.9) years. Clinical relapses occurred in 30% (9 of 30) during rituximab treatments. The relapses coincided with B cell depletion in 8 of 9, including returning of multiorgan symptoms (*n* = 4), localized disease flare such as scleritis (*n* = 2), or interstitial lung disease (ILD) progression (*n* = 2); and B cell repopulation in 1 of 9 patients who had sinopulmonary disease. The relapses were treated with repeat rituximab infusions and/or other additional immunosuppression.

### Outcomes Features After the Date of the Last Rituximab Dose

#### Duration of B Cell Depletion and B Cell Repopulation

**Figure 1:**
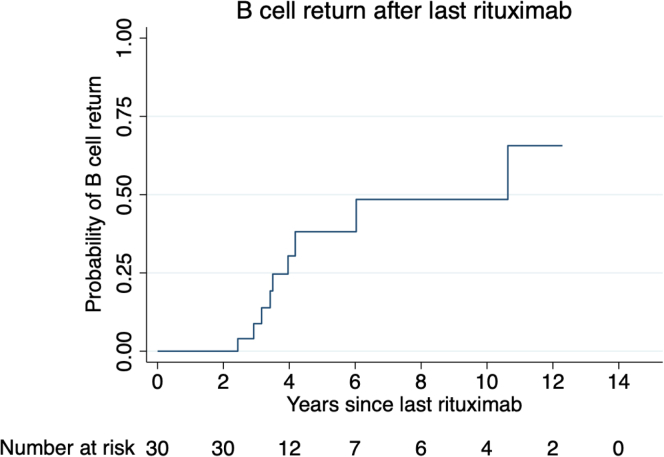
Kaplan-Meier plot of B cell return following persistent B cell depletion after the last rituximab infusion.

The median follow-up after the last rituximab dose was 4.1 (IQR: 2.6–6.0) years. The cumulative incidence of B cell repopulation (> 5 cells/μl) was 30% and 48% at 4 and 8 years, respectively, after the last rituximab dose (Figure 1). In patients with B cell repopulation, the B cell counts remained very low, at a median of only 7 (6–15) cells/μl at the last follow-up. None of the patients were retreated with rituximab after B cell repopulation.

#### Disease Activity After the Last Rituximab Dose

The outcomes after the last rituximab dose are summarized in Table 2. After the last rituximab dose, 83% (25 of 30) of the patients had sustained remission from their primary disease. Serum ANCA titers remained low in patients with AAV (Table 2). Long-term maintenance steroids were used in 37% (11 of 30). Only 17% (5 of 30) had disease activity, which occurred during B cell depletion and received further immunosuppression (Table 2). Two patients with ANCA-associated ILD had progression of their ILD despite suppressed ANCA titers. Another patient with AAV experienced recurrent episodes of scleritis and nasal crusting despite suppressed ANCA titers. One of the patients with ANCA-negative vasculitis, who had recurrent uveitis, scleritis, and skin purpura despite multiple courses of cytotoxic therapies, partially responded to rituximab and continued to have flares of localized disease with scleritis. Lastly, the patient with Behcet’s disease, who failed to respond to many lines of other immunosuppression, also failed to respond to rituximab.

**Table 2:** Clinical features after the last rituximab dose (*n* = 30)

Clinical features	Median (IQR) or *n* (%)
Follow-up duration since last rituximab dose(yrs)	4.1 (2.6–6.0)
Patients who achieved sustained remission	25 (83)
Patients who experienced disease activity	5 (17)
ILD progression despite suppression of ANCA	2 (40)
AAV symptoms despite suppression of ANCA	1 (20)
ANCA-negative vasculitis	1 (20)
Behcet’s disease	1 (20)
ANCA titers at last follow-up among patients with AAV	
MPO ANCA (reference: <2.8 U)	2.3 (1.0–5.1)
PR3 ANCA (reference: <20 U)	2.2 (1.1–4.1)
Long-term maintenance steroids	11 (37)
Adverse events after the last rituximab dose	
Adrenal insufficiency	10 (33)
Late-onset neutropenia of rituximab	7 (23)
Hypogammaglobulinemia	29 (97)
Nadir IgG (reference: > 614 mg/dl)	447 (319–605)
Nadir IgM (reference: > 53 mg/dl)	21 (9–26)
Nadir IgA (reference: > 69 mg/dl)	73 (28–123)
Recurrent infections	14 (47)
Recurrent lower respiratory tract infections	6 (20)
Recurrent upper respiratory tract infections	5 (17)
Recurrent urinary tract infections	3 (10)
Recurrent otitis	2 (7)
Recurrent gastrointestinal infections	1 (3)
Chronic bronchitis and/or cough	12 (40)
Chronic inflammatory vaginitis	3 (18)
Severe infections	17 (57)
Bacterial or viral pneumonia (excluding COVID-19)	6 (20)
Prolonged or severe COVID-19 pneumonia	3 (10)
Aspergillus pneumonia	1 (3)
Urinary tract infection	4 (13)
Febrile neutropenia	2 (7)
Varicella zoster	2 (7)
Syphilis	1 (3)
Peritoneal dialysis catheter-associated peritonitis	1 (3)
Clostridium difficile colitis	1 (3)
Diabetic wound infection and osteomyelitis	1 (3)
IgG replacement	8 (23)
Malignancy	4 (13)
Breast cancer	1 (25)
Prostate cancer	1 (25)
Metastatic melanoma	1 (25)
Natural killer cell leukemia	1 (25)
Death	9 (30)
Respiratory failure because of ANCA-associated ILD	2 (22)
Respiratory failure because of severe COPD	2 (22)
Acute legionella pneumonia	1 (11)
Acute COVID-19 pneumonia	1 (11)
Febrile neutropenia	1 (11)
Congestive heart failure	1 (11)
End-stage dementia	1 (11)

AAV, ANCA-associated vasculitis; ANCA, antineutrophil cytoplasmic autoantibody; COPD, chronic obstructive pulmonary disease; ILD, interstitial lung disease; IQR, interquartile range; MPO, myeloperoxidase; PR3, proteinase 3.

#### Adverse Events After the Last Rituximab Dose

The adverse events are summarized in Table 2. Adrenal insufficiency was present or occurred in 33% (10 of 30). Late-onset neutropenia occurred in 23% (7 of 30), all with AAV. The median time from the last rituximab dose to the latest episode of late-onset neutropenia was 0.4 (IQR: 0.2–3.3) years. Five of 7 patients who had late-onset neutropenia experienced neutropenic fever or infection. One patient had recurrent and severe neutropenia episodes starting 18 months before the last rituximab dose and later became dependent on and ultimately resistant to injections of recombinant human granulocyte colony-stimulating factor. Bone marrow biopsy of this patient showed no B cells, 26% neutrophil or neutrophil precursors, and a decreased myeloid-to-erythroid ratio and myeloid maturation when the patient had no B cells and no neutrophils in peripheral blood. The patient died from a venous port infection during a neutropenia episode at 4.6 years after the last rituximab dose.

Hypogammaglobulinemia occurred in all but 1 patient. The median baseline IgG level before rituximab initiation was 837 (IQR: 691–1030), similar to that of patients who eventually had B cell repopulation versus those who did not (*P* = 0.567). IgG reached a nadir at a median of 2 (IQR: −0.2 to 4.5) years after the last rituximab dose. Nadir serum IgG, IgM, and IgA levels are depicted in Table 2. Nadir IgG levels were < 400 mg/dl in 40% (12 of 30) and < 300 mg/dl in 20% (6 of 30). The median IgG level remained low at 485 (IQR: 366–646) mg/dl in patients with persistent B cell depletion at the last follow-up, after excluding patients who received Ig replacement. In patients who had B cell repopulation, IgG levels were similar immediately before B cell repopulation and at the last follow-up (708 [IQR: 560–907] vs. 798 [IQR: 511–1120] mg/dl, *P* = 0.936).

Recurrent infectious episodes occurred in 47% (14 of 30), which included mostly upper and lower respiratory tract infections (Table 2). Nadir IgG levels were similar in patients who developed recurrent infections and those who did not (447 [IQR: 275–615] vs. 451 [IQR: 340–584] mg/dl, respectively; *P* = 0.609). Chronic bronchitis occurred in 20% (6 of 30) and chronic inflammatory vaginitis in 18% (3 of 17) of female patients. Severe infections requiring i.v. antibiotics or hospitalization occurred in 57% (17 of 30) of patients (Table 2). Of the patients, 23% (8 of 30) were started on Ig replacement therapy for infection (*n* = 7), chronic bronchitis (*n* = 6), and/or inflammatory vaginitis (*n* = 1). IgG replacement led to symptom improvement in all patients and continued until the last follow-up, except for 1 patient. Cancer complicated the course in 13% (4 of 30) of patients (Table 2).

A total of 9 patients (30%) died at a median age of 74 (IQR: 63–84) years at 4.3 (IQR: 2.8–7.0) years after the last rituximab dose. The deaths were attributed to the complications of respiratory failure because of ANCA-associated ILD in 2 patients, infections in 3 patients, and complications of other chronic diseases in 4 patients (Table 2).

## Discussion

Here, we describe a new phenomenon, “persistent B cell depletion,” as a rare complication of rituximab treatment that occurred in 2% of patients with autoimmune and glomerular diseases on B cell depletion with rituximab. Patients who developed persistent B cell depletion typically had a prolonged and recurrent underlying disease, necessitating high cumulative doses of cytotoxic therapies. Persistent B cell depletion after rituximab treatment was characterized by a quiescent disease course at the expense of complications of B cell depletion, including recurrent infections, inflammatory vaginitis, and late-onset neutropenia. Ig replacement was key in controlling infectious or inflammatory complications of persistent B cell depletion in one-third of the patients.

Previous studies have shown several risk factors for prolonged B cell depletion after rituximab treatment. The time to B cell reconstitution after rituximab treatment was longer in patients with AAV, aged > 60 years and with estimated glomerular filtration rate < 30 ml/min per 1.73 m^2^.^18^^,^^19^^,^^23^^,^^24^ In our study, the frequency of persistent B cell depletion was similar in AAV and lupus nephritis but numerically lower in other diagnoses such as minimal change disease or focal segmental glomerulosclerosis and membranous nephropathy. Our patients' median age of 63 years was consistent with an increased risk for prolonged B cell depletion; however, their kidney function was relatively well-preserved. Given their suppressive effects on B cells, it is plausible to think that concurrent exposure to cytotoxic immunosuppressants prolongs B cell depletion after rituximab; however, the data are inconsistent.^19^^,^^20^^,^^24^ In a cohort of 839 adult patients with various autoimmune diseases and solid organ transplantation receiving rituximab, the duration of B cell depletion was 133 and 193 days longer in patients exposed to corticosteroids and azathioprine, respectively.^24^ However, a similar correlation was not observed in patients exposed to cyclophosphamide and mycophenolate mofetil in the same study. In pediatric patients with autoimmune diseases, B cell depletion > 12 months tended to occur more often in patients receiving concurrent cyclophosphamide (16% vs. 4%).^20^ Conversely, no correlation was found between the cumulative cyclophosphamide dose and the duration of B cell depletion in a cohort of adult patients with AAV, rheumatoid arthritis, and connective tissue disorder.^19^ Most of our patients had received more prolonged and recycling cytotoxic agents and maintenance steroids for their recurrent diseases compared with a typical patient with the same diagnosis, who usually has B cell repopulation within 6 to 12 months.^13^ Moreover, none of the patients with membranous nephropathy developed persistent B cell depletion, a population usually exposed to less immunosuppression.^10^ Thus, we suspect that high exposure to cytotoxic immunosuppressants for recurrent diseases played a significant role in persistent B cell depletion in our cohort. Lastly, none of the studies showed a correlation between the number of rituximab doses and the duration of B cell depletion.^18^^,^^20^^,^^24^ The report of 2 patients with SLE who developed persistent B cell depletion > 5 years after only a single cycle of rituximab supports this.^21^

Rituximab targets CD20 and kills B cells diffusely, including autoreactive and nonreactive B cells, predominantly by a complement-mediated mechanism.^25^ Because B cell progenitors lack CD20 expression, they serve as a source of reconstitution; thus, B cell depletion after rituximab is usually not permanent. Moreover, although rituximab depletes peripheral B cells completely, it usually induces only partial B cell depletion in secondary lymphoid organs and other tissues, which helps reconstitute the peripheral B cell pool.^12^^,^^26^, ^27^, ^28^, ^29^, ^30^ Despite these protective factors, the reason why some patients develop persistent B cell depletion after rituximab is not fully known. A bone marrow biopsy of 1 of our patients (#6) showed no B cells despite a relatively normal marrow otherwise, similar to that of a patient with SLE with persistent B cell depletion,^21^ postulating a decreased bone marrow B cell reserve. It can be speculated that, in addition to rituximab itself, concomitant cytotoxic agents and potentially underlying relapsing diseases and recurrent infections played a role in delaying the reconstitution of B cells in our patients. Moreover, a recent study showed that defects in B-lymphopoiesis in the bone marrow, including low transitional B cell count and under expression of B cell activating factor–receptor by B cells, contribute to prolonged B cell depletion.^31^ Further mechanistic studies are needed to elucidate this rare complication. In the future, developing strategies that target only autoreactive B cells rather than killing B cells diffusely may help prevent this rare complication.^32^

Most of our patients with persistent B cell depletion remained in remission during persistent B cell depletion after the last rituximab dose. Earlier, we showed that the repopulation of B cells poses a risk for disease relapse in AAV.^13^ Moreover, a faster B cell repletion after rituximab was associated with a higher risk of relapse.^18^ However, relapse can also occur during peripheral B cell depletion.^18^ Indeed, nearly 30% of our patients had ≥ 1 relapse during full B cell depletion while receiving rituximab infusions, likely reflecting the relapsing nature of this specific population and the contribution of other pathogenic mechanisms. In lupus nephritis, achieving full B cell depletion was key to achieving complete remission.^11^ In rheumatoid arthritis, symptoms recur after B cell repopulation in patients treated with rituximab.^15^ Thus, persistent B cell depletion likely played a key role in maintaining disease remission in our patients. Two patients with ANCA-associated ILD had ILD progression despite suppression of B cells and ANCA titers. However, by unknown mechanisms, this group of patients is known to have a progressive course and increased mortality even in the absence of residual or recurrent ANCA.^33^ Lastly, although the depletion of CD5^+^ regulatory B cells may portend a risk for relapse in AAV, this risk is less applicable to patients with complete B cell depletion, likely because of the absence of effector B cells.^34^^,^^35^

Not surprisingly, almost all our patients had hypogammaglobulinemia, and half of them had recurrent and/or severe infections, which postulates a higher complication rate compared with patients who do not develop persistent B cell depletion.^13^ In our previous study, serum IgG levels < 400 mg/dl were associated with an increased risk of serious infection after rituximab,^36^ which occurred in 39% of our cohort. Therefore, ongoing monitoring for infections and inflammatory events is paramount in these patients. The success of Ig replacement in our patients with infectious and inflammatory complications, such as chronic or relapsing bronchitis and inflammatory vaginitis, postulates the utility of this treatment, which needs to be confirmed in larger studies. These considerations are especially important with COVID-19 infections, because these patients have a blunted immune response to previous infections and vaccines. Although the death rate was relatively high in our cohort, most deaths occurred because of underlying severe lung disease with accruing lung damage and other comorbidities rather than infections (Table 2).

Late-onset neutropenia occurred in 23% of our patients, which is more frequent than expected.^37^^,^^38^ Our previous study showed that late-onset neutropenia after rituximab is more common in patients with SLE and concomitant exposure to cyclophosphamide, and mostly occurs within the first year following rituximab initiation.^37^ In contrast, in this study, all patients who developed late-onset neutropenia had AAV, and none had SLE. Episodes of late-onset neutropenia continued long after the first year of rituximab initiation. The higher incidence and delayed onset of late-onset neutropenia in our cohort might implicate 2 factors as follows: (i) high exposure to cytotoxic agents, including cyclophosphamide, which can induce neutropenia and (ii) persistent B cell depletion causing cytokine imbalances, such as B cell activating factor and stromal-derived factor 1, that might inhibit neutrophil production or egression. Intriguingly, patient #6, who experienced recurrent, severe, and intractable episodes of late-onset neutropenia, had decent neutrophils and their precursors in the bone marrow, similar to a previous report,^38^ suggesting the inhibition of neutrophil egression from bone marrow.

Our study has certain limitations. First, because this was not a case-control study, we only reported the frequency of persistent B cell depletion and clinical features of the patients and could not determine the risk factors by adjusting for multiple variables. Yet, this study still provides key insights to clinicians about the characteristics and management of patients with persistent B cell depletion after rituximab. Second, although we provided the number of cycles and the type of concurrent or previous immunosuppressive agents, we could not quantify the cumulative dose of each agent, given inconsistent chart data. Lastly, the patient population at our center had more patients with AAV and autoimmune kidney diseases and fewer other autoimmune diseases such as rheumatoid arthritis. Thus, our data must be confirmed in a more diverse patient population.

In conclusion, persistent B cell depletion is a rare complication of rituximab treatment that is associated with maintained remissions but at the cost of increased risk of complications of B cell depletion. Clinicians should discuss the risk of persistent B cell depletion with their patients when prescribing rituximab, especially when they have had recurrent disease and high exposure to cytotoxic immunosuppressants. This complication might be observed less often with the use of rituximab as a first-line therapy and, thus, avoiding high exposure to cytotoxic immunosuppression in certain autoimmune conditions. Future prospective studies confirming our findings and mechanistic studies exploring the underpinnings of this complication are needed.

## Disclosure

FBC has received consulting and speaking fees for Amgen, Aurinia, Travere, and Calliditas. RZ has received consulting and speaker fees from Amgen and Novartis. AJ receives honoraria from Calliditas Therapeutics. JLN receives grants from Amgen, outside the submitted work. All the other authors declared no conflicting interests.

## Supplementary Material

Supplementary File (PDF) **Table S1.** Patient diagnosis among all patients who received rituximab. **Table S2.** Individual characteristics of cases with persistent B cell depletion after rituximab.

## Footnotes

Supplementary File (PDF)
**Table S1.** Patient diagnosis among all patients who received rituximab. **Table S2.** Individual characteristics of cases with persistent B cell depletion after rituximab.
