# Supplementary material for: Persistent B Cell Depletion After Rituximab for Autoimmune and Glomerular Diseases: A Case Series
Source: Kidney Int Rep. 2025 Feb 7;10(5):1441–9. doi: 10.1016/j.ekir.2025.02.002 (PMC12142800; doi:10.1016/j.ekir.2025.02.002)
Supplement: Supplementary File (PDF) — Table S1. Patient diagnosis among all patients who received rituximab. Table S2. Individual characteristics of cases with persistent B cell depletion after rituximab. [file mmc1.pdf]

# Persistent B cell depletion after Rituximab for autoimmune and glomerular diseases: a case series

## Supplementary Materials

**Table S1. Patient diagnosis among all patients who received rituximab.**

| Diagnosis | Frequency | Percent |
|---|---|---|
| ANCA-associated vasculitis | 878 | 57.8 |
| Membranous nephropathy | 133 | 8.76 |
| Minimal change disease / focal segmental glomerulosclerosis | 114 | 7.5 |
| Systemic lupus erythematosus / lupus nephritis | 82 | 5.4 |
| ANCA-negative vasculitis | 37 | 2.44 |
| Autoimmune interstitial lung disease (uncategorized) | 28 | 1.84 |
| Eosinophilic granulomatosis with polyangiitis (EGPA) | 27 | 1.78 |
| Rheumatoid arthritis | 27 | 1.78 |
| IgG-4-related disease | 27 | 1.78 |
| Dermatomyositis | 22 | 1.45 |
| Cryoglobulinemia | 16 | 1.05 |
| Fibrillary glomerulonephritis | 16 | 1.05 |
| Polymyositis | 14 | 0.92 |
| Systemic lupus erythematosus | 13 | 0.86 |
| Anti-GBM disease and ANCA-associated vasculitis | 10 | 0.66 |
| Membranoproliferative glomerulonephritis | 9 | 0.59 |
| Sjogren's Disease | 7 | 0.46 |
| Autoimmune retinopathy / retinal vasculitis | 7 | 0.46 |
| Anti-GBM disease | 6 | 0.39 |
| IgA nephropathy | 6 | 0.39 |
| Polyarteritis nodosa | 6 | 0.39 |
| Anti-phospholipid antibody syndrome | 5 | 0.33 |
| Monoclonal gammopathy | 5 | 0.33 |
| Bullous pemphigoid | 4 | 0.26 |
| Humoral transplant rejection | 4 | 0.26 |
| Mixed connective tissue disorder | 4 | 0.26 |
| C3 glomerulopathy | 3 | 0.2 |
| Thrombotic microangiopathy | 3 | 0.2 |
| ANCA-vasculitis and fibrillary glomerulonephritis | 2 | 0.13 |
| Polymyositis | 2 | 0.13 |

| | | |
|---|---|---|
| Relapsing polychondritis | 2 | 0.13 |
| Acute disseminating encephalomyelopathy (ADEM) | 1 | 0.07 |
| Anti-GBM Disease | 1 | 0.07 |
| Autoimmune Hepatitis | 1 | 0.07 |
| Behcet's Disease | 1 | 0.07 |
| Graves ophthalmopathy | 1 | 0.07 |
| Myasthenia Gravis | 1 | 0.07 |
| Myositis (uncategorized) | 1 | 0.07 |
| Neuromyelitis Optica | 1 | 0.07 |
| Retroperitoneal Fibrosis | 1 | 0.07 |
| Stiff Persons Syndrome | 1 | 0.07 |
| Susac's Syndrome | 1 | 0.07 |
| Takayasu's arteritis | 1 | 0.07 |
| Hypereosinophilic syndrome | 1 | 0.07 |
| **Total** | **1,519** | **100** |

# Table S2.Individual characteristics of cases with persistent B cell depletion after rituximab.

| Study ID | Diagnosis | Age | Gender | History of other autoimmune diseases and hematological malignancies | Immunosuppression preceding or concurrent with rituximab initiation | Long-term maintenance steroids preceding or concurrent with rituximab initiation | Rituximab exposure (cumulative dose) | Duration of B-cell depletion since last RTX dose (years) | Duration of follow-up post-last RTX (years) | Disease relapse after last rituximab | Serum IgG level (nadir) | Ig replacement therapy | Recurrent infections | Severe infections | LON episodes |
|---|---|---|---|---|---|---|---|---|---|---|---|---|---|---|---|
| 1 | LN | 33 | F | Juvenile rheumatoid arthritis | AZA for >1y, MMF for >7y, ATG induction and tacrolimus maintenance for kidney transplant | yes | 7000 | 12.3 | 12.3 | no | <40 | yes | yes | Yes | no |
| 2 | MPO AAV | 51 | M | none | Multiple cycles of CYC, MMF and AZA for many years | yes | 12000 | 10.0 | 10.0 | no | 320 | yes | yes | yes | no |
| 3 | MPO AAV | 49 | M | Inflammatory bowel disease, NK cell lymphocytosis | One cycle of CYC, AZA for ~6 months, MTX for >3y | yes | 20940 | 8.8 | 8.8 | no | 468 | yes | yes | yes | no |
| 4 | PR3 AAV | 36 | M | Inflammatory bowel disease | CYC and steroids for 26 years, AZA for many years, MMF for >8y, MTX for ~1y | yes | 20337 | 11.3 | 11.3 | no | 287 | no | yes | yes | no |
| 5 | MPO AAV | 27 | F | None | AZA maintenance for >5y | yes | 5000 | 10.6 | 10.6 | no | 240 | yes | yes | yes | yes |
| 6 | MPO AAV | 60 | F | None | 2 cycles of CYC, and AZA for >6y | no | 8000 | 4.0 | 4.3 | no | 356 | yes | no | yes | yes |
| 7 | PR3 AAV | 47 | F | None | MMF for >7y | no | 11000 | 2.9 | 10.0 | no | 635 | no | yes | yes | yes |
| 8 | PR3 AAV | 69 | M | None | AZA and MMF maintenance for kidney transplant for many years | yes | 12000 | 3.5 | 8.0 | no | 315 | no | no | yes | yes |
| 9 | MPO AAV | 81 | F | None | One cycle of CYC | yes | 19000 | 2.8 | 2.8 | no | 485 | no | no | no | no |
| 10 | MPO AAV | 53 | F | CML | One cycle of CYC | yes | 9000 | 5.6 | 5.6 | no | 233 | no | no | no | no |
| 11 | Minimal Change disease / FSGS | 67 | M | None | MMF for ~3y | yes | 20000 | 2.5 | 2.5 | no | 335 | no | no | no | no |
| 12 | MPO AAV | 47 | F | None | Two cycles of CYC and AZA maintenance for many years | yes | 10000 | 3.2 | 3.2 | no | 746 | no | yes | yes | yes |
| 13 | MPO AAV | 78 | M | MGUS | None | yes | 12000 | 6.0 | 6.0 | no | 604 | no | no | no | no |
| 14 | MPO AAV | 79 | M | None | Two cycles of CYC | no | 14000 | 2.4 | 4.5 | no | 511 | no | no | no | no |
| 15 | MPO AAV | 31 | F | None | Multiple cycles of CYC, AZA for ~2y | yes | 15000 | 4.0 | 5.1 | no | 761 | no | no | no | yes |
| 16 | ANCA-negative vasculitis | 71 | F | None | Two cycles of CYC | yes | 12000 | 4.5 | 4.7 | no | 421 | no | yes | no | no |
| 17 | MPO AAV | 79 | M | None | One cycle of CYC | no | 6000 | 2.6 | 2.6 | no | 240 | yes | yes | yes | yes |
| 18 | MPO AAV | 68 | M | None | One cycle of CYC | yes | 11000 | 4.0 | 4.0 | ILD progressi on | 433 | no | yes | yes | no |
| 19 | MPO AAV | 78 | F | None | One cycle of CYC | yes | 8660 | 3.4 | 3.4 | no | 775 | no | yes | yes | no |
| 20 | MPO AAV | 74 | M | None | One cycle of CYC | no | 9000 | 3.2 | 3.2 | no | 819 | no | no | no | no |
| 21 | MPO AAV | 76 | M | Anti-GBM positive | One cycle of CYC and plasma exchange x7 | no | 12000 | 2.2 | 2.2 | no | 389 | no | no | no | no |
| 22 | ANCA-negative vasculitis | 26 | F | None | CYC for 9 months and AZA for >3y | yes | 11000 | 2.6 | 2.6 | Recurrent scleritis | 461 | yes | yes | yes | no |
| 23 | MPO AAV | 71 | F | Anti-GBM positive | One cycle of CYC | no | 8000 | 4.1 | 4.1 | no | 197 | no | no | no | no |
| 24 | LN | 20 | F | Autoimmune diabetes and autoimmune hepatitis | One course of CYC, ATG induction and CysA for aplastic anemia | no | 13000 | 2.9 | 2.9 | no | 522 | no | no | yes | no |
| 25 | Behcet's Disease | 56 | F | None | Alternating AZA, MMF, anti-TNF, CYC, CysA, tocilizumab for many years | yes | 4000 | 2.4 | 2.4 | Failed remission | 485 | no | no | yes | no |
| 26 | MPO AAV | 37 | F | Psoriasis and psoriatic arthritis | One cycle of CYC | no | 9000 | 4.0 | 4.0 | no | 812 | no | no | no | no |
| 27 | PR3 AAV | 26 | F | None | One cycle of CYC, AZA and MMF for >4 years, avacopan for >2 years | yes | 8000 | 4.2 | 4.2 | Recurrent scleritis | 471 | yes | yes | yes | no |
| 28 | Polymyositis | 68 | M | None | MMF for >5 years | yes | 8000 | 2.4 | 2.4 | no | 608 | no | yes | yes | no |
| 29 | MPO AAV | 75 | M | None | Three cycles of CYC, AZA for >3y, avacopan for 1y | yes | 7000 | 2.3 | 2.3 | ILD progressi on | 417 | no | no | no | no |

| | | | | | | | | | | | | | | | |
|---|---|---|---|---|---|---|---|---|---|---|---|---|---|---|---|
| 30 | Anti-GBM disease | 65 | F | None | None | no | 3000 | 2.0 | 2.0 | no | 382 | no | no | no | no |

AAV, ANCA-associated vasculitis; anti-GBM, anti-glomerular basement membrane disease; ATG, anti-thymocyte globulin; AZA, azathioprine; CYC, cyclophosphamide; CysA, cyclosporin; F, female; ILD, interstitial lung disease; LN, lupus nephritis; LON, late onset neutropenia; M, male; MMF, mycophenolate mofetil; PLEX; plasma exchange; TNF, tumor necrosis factor
